# Supplementary material for: Assessing risk factors and impact of cyberbullying victimization among university students in Myanmar: A cross-sectional study
Source: PLoS One. 2020 Jan 22;15(1):e0227051. doi: 10.1371/journal.pone.0227051 (PMC6975531; doi:10.1371/journal.pone.0227051)
Supplement: S1 Questionnaire — (PDF) [file pone.0227051.s001.pdf]

ကျောင်းသား၊ ကျောင်းသူများထံမှ နည်းပညာ (လက်ကိုင်ဖုန်း၊ ဖေ့စ်ဘုတ်၊ လူမှုကွန်ယက်၊ အင်တာနက်၊ လျှို့ဝှက်ကင်မရာ) များအသုံးပြု၍ ထိခိုက် နစ်နာစေမှု နှင့် ဆက်စပ်သည့် အချက်အလက်များကို လေ့လာသည့် မေးခွန်းလွှာများသည် သုတေသန ပြုလုပ်ရန်အတွက်သာ ဖြစ်သည်။ အဖြေလွှာများ ကို စနစ်တကျ၊ လုံခြုံစွာ ထိန်းသိမ်းထားမည် ဖြစ်သောကြောင့် လွတ်လပ်စွာ ဖြေဆိုနိုင်ပါသည်။

နေ့စွဲ ____________________ အမှတ်စဉ် | | | |

အပိုင်း (၁) ဖြေဆိုသူ၏ နောက်ခံ အချက်အလက်များ (သင်ဖြေဆိုလိုသည့် အဖြေ၏ နံပါတ်ကို ဝိုင်းပါ။)

စဉ်	မေးခွန်း	အဖြေ
၁။	အသက် (ပြည့်ပြီးအသက်)	____________________ နှစ်
၂။	ကျား/မ	၁။ ကျား<br>၂။ မ
၃။	လက်ရှိအိမ်ထောင်ရေးအခြေအနေ	၁။ လူပျို၊ အပျို<br>၂။ အိမ်ထောင်ရှိ<br>၃။ ကွာရှင်း/အတူမနေ (ကွဲကွာ)<br>၄။ မုဆိုးဖို/ မုဆိုးမ
၄။	ဤတက္ကသိုလ်တွင် ယနေ့အချိန်အထိပညာသင်ကြားနေသော အချိန် (နှစ်)စုစုပေါင်း	______________________(နှစ်)
၅။	သင်ယခု ကျောင်းတက်နေစဉ် မိသားစုနှင့်ခွဲ၍ အဆောင်နေပါသလား။	၁။ အဆောင်နေပါသည်။<br>၂။ အဆောင်မနေပါ။
၆။	သင့်အိမ်တည်ရှိသော တိုင်းဒေသကြီး၊ ပြည်နယ်	__________________________
၇။	သင်လတ်တလောအများဆုံး အသုံးပြုနေသော လူမှုကွန်ယက်များမှာ<br>(အဖြေတစ်ခုထက်ပို၍ ဖြေဆိုနိုင်ပါသည်။)	၁. facebook<br>၂. Instagram<br>၃. You tube<br>၄. Viber<br>၅. We Chat<br>၆. Bee Talk<br>၇. အခြား ________________<br>(တိတိကျကျဖော်ပြပါ။)
၈။	လူမှုကွန်ယက်များပေါ်တွင် သင်တနေ့ပျမ်းမျှ အချိန် မည်မျှသုံးပါသလဲ။	____________(မိနစ်/နာရီ)

အပိုင်း (၂) မေးခွန်း
နည်းပညာ (လက်ကိုင်ဖုန်း၊ ဖေ့စ်ဘုတ်၊ လူမှုကွန်ယက်၊ အင်တာနက်၊ လျှို့ဝှက်ကင်မရာ) များအသုံးပြု၍ ထိခိုက် နစ်နာစေမှု
(သင်ဖြေဆိုလိုသည့် အဖြေ၏ နံပါတ်ကို ဝိုင်းပါ။)

စဉ်	မေးခွန်း	အဖြေ
	**တစ်စုံတစ်ယောက်သည် သင့်အား အောက်ဖော်ပြပါအချက်များကို ပြုလုပ်ခဲ့ဖူးပါသလား။**	
၁။	တစ်စုံတစ်ယောက်သည် သင့်လက်ကိုင်ဖုန်း၊ လူမှုကွန်ယက် များကို ခွင့်ပြုချက်မရှိဘဲ စစ်ဆေးခြင်း (သို့) ခိုးယူအသုံးပြု ခြင်း (သို့) သင့် Password/လူမှုကွန်ယက်အကောင့်များ ကို ခိုးယူအသုံးပြု၍ သင့်ဟန်ဆောင်ခြင်းကို ပြီးခဲ့တဲ့ (၁၂) လ အတွင်း ကြုံတွေ့ခဲ့ဖူးပါသလား။	၁။ ကြုံတွေ့ခဲ့ရပါသည်။<br>၂။ မကြုံတွေ့ခဲ့ရပါ။<br>၃။ မသိပါ။<br>၄။ မဖြေဆိုလိုပါ။
၂။	တစ်စုံတစ်ယောက်သည် သင့်ခွင့်ပြုချက်မပါဘဲ သင့်ဓာတ်ပုံများအား အွန်လိုင်းပေါ်တင်ခြင်း (သို့) အွန်လိုင်းပေါ်တွင်အသုံးပြုခြင်းကို ပြီးခဲ့တဲ့ (၁၂) လ အတွင်း ကြုံတွေ့ခဲ့ရဖူးပါသလား။	၁။ ကြုံတွေ့ခဲ့ရပါသည်။<br>၂။ မကြုံတွေ့ခဲ့ရပါ။<br>၃။ မသိပါ။<br>၄။ မဖြေဆိုလိုပါ။
၃။	တစ်စုံတစ်ယောက်က အွန်လိုင်း/လူမှုကွန်ယက်များမှတစ်ဆင့် သင်နှင့်ပတ်သက် သောမဟုတ်မမှန် သတင်းများ (သို့) ကောလဟာလများလွှင့်ခြင်းကို ပြီးခဲ့တဲ့ (၁၂) လ အတွင်း ကြုံတွေ့ခဲ့ရဖူးပါသလား။	၁။ ကြုံတွေ့ခဲ့ရပါသည်။<br>၂။ မကြုံတွေ့ခဲ့ရပါ။<br>၃။ မသိပါ။<br>၄။ မဖြေဆိုလိုပါ။
၄။	တစ်စုံတစ်ယောက်က သင့်အားအရှက်ရစေသော၊ စိတ်အနှောင့်အယှက်ဖြစ်စေသော၊ ရိုင်းစိုင်းသော စာသားများ၊ အကြောင်း အရာများ (သို့) လိင်မှုကိစ္စများနှင့်သက်ဆိုင်သော စကားများပေးပို့ခြင်းကို ပြီးခဲ့တဲ့ (၁၂) လ အတွင်း ကြုံတွေ့ခဲ့ရဖူးပါသလား။	၁။ ကြုံတွေ့ခဲ့ရပါသည်။<br>၂။ မကြုံတွေ့ခဲ့ရပါ။<br>၃။ မသိပါ။<br>၄။ မဖြေဆိုလိုပါ။
၅။	တစ်စုံတစ်ယောက်က သင့်ကိုအနှောင့်အယှက်ဖြစ်စေသည့် ဖုန်းခေါ်ဆိုခြင်းများ(သို့) ဖုန်းပြောဆိုခြင်းများကို ပြီးခဲ့တဲ့ (၁၂) လ အတွင်း ကြုံတွေ့ခဲ့ ရဖူးပါသလား။	၁။ ကြုံတွေ့ခဲ့ရပါသည်။<br>၂။ မကြုံတွေ့ခဲ့ရပါ။<br>၃။ မသိပါ။<br>၄။ မဖြေဆိုလိုပါ။
၆။	တစ်စုံတစ်ယောက်က သင့်သဘောဆန္ဒမပါဘဲ သင့်ထံသို့ ဆိုးရွားသည့် ဓာတ်ပုံများ(သို့)ဗွီဒီယိုများ(သို့)ညစ်ညမ်းပုံများ ပေးပို့ခြင်းကို ပြီးခဲ့တဲ့ (၁၂) လ အတွင်း ကြုံတွေ့ခဲ့ရဖူးပါသလား။	၁။ ကြုံတွေ့ခဲ့ရပါသည်။<br>၂။ မကြုံတွေ့ခဲ့ရပါ။<br>၃။ မသိပါ။<br>၄။ မဖြေဆိုလိုပါ။

၇။	တစ်စုံတစ်ယောက်က သင့်ခွင့်ပြုချက်မပါဘဲ ခိုးဝှက်၍ သင့်ဓါတ်ပုံ၊ ဗွီဒီယိုများရိုက်ကူးခြင်း (သို့) ရိုက်ကူး၍ အရှက်ခွဲခြင်း (သို့) ခြိမ်းခြောက်ခြင်း၊ ငွေညှစ်ခြင်းကို ပြီးခဲ့တဲ့ (၁၂) လ အတွင်း ကြုံတွေ့ခဲ့ရဖူးပါသလား။	၁။ ကြုံတွေ့ခဲ့ရပါသည်။<br>၂။ မကြုံတွေ့ခဲ့ရပါ။<br>၃။ မသိပါ။<br>၄။ မဖြေဆိုလိုပါ။
၈။	အထက်ပါနည်းပညာအသုံးပြု၍ ထိခိုက်နစ်နာစေမှု တစ်မျိုးမျိုးကို ပထမဆုံးစတင်ကြုံတွေ့ရစဉ်က သင့်အသက် (ပြည့်ပြီးအသက်) ကိုဖော်ပြပါ။	---------------နှစ်
၉။	သင်တစ်ယောက်ယောက်ကို အထက်ပါထိခိုက်နစ်နာ ခံစားရမှု (များ)နှင့် ပတ်သက်ပြီး ပြောပြခဲ့ဖူးပါသလား။	၁။ ပြောပြခဲ့ဖူးပါသည်။<br>၂။ မပြောပြခဲ့ဖူးပါ။<br>၃။ မသိပါ။<br>၄။ မဖြေဆိုလိုပါ။

အပိုင်း (၃) မေးခွန်း

(က) နည်းပညာ (လက်ကိုင်ဖုန်း၊ ဖေ့စ်ဘုတ်၊ လူမှုကွန်ယက်၊ အင်တာနက်၊ လျှို့ဝှက်ကင်မရာ များအသုံးပြု ထိခိုက်နစ်နာ မှုကြောင့် အကျိုးသက်ရောက်မှု (သင်ဖြေဆိုလိုသည့် အဖြေ၏ နံပါတ်ကို ဝိုင်းပါ။)

စဉ်	မေးခွန်း	အဖြေ
၁။	အထက်ပါ ထိခိုက်နစ်နာစေမှု(များ)ကြောင့် သင်ဆေးလိပ်စသောက်တတ်လာသည် (သို့) ပိုသောက်လာသည်။	၁။ ဟုတ်ပါသည်။<br>၂။ မဟုတ်ပါ။<br>၃။ မသိပါ။<br>၄။ မဖြေဆိုလိုပါ။
၂။	အထက်ပါ ထိခိုက်နစ်နာစေမှု(များ)ကြောင့် သင် ကွမ်း စစားတတ်လာသည် (သို့) ပိုစားလာသည်။	၁။ ဟုတ်ပါသည်။<br>၂။ မဟုတ်ပါ။<br>၃။ မသိပါ။<br>၄။ မဖြေဆိုလိုပါ။
၃။	အထက်ပါ ထိခိုက်နစ်နာစေမှု(များ)ကြောင့် သင်အရက်စသောက်တတ်လာသည် (သို့) ပိုသောက်လာသည်။	၁။ ဟုတ်ပါသည်။<br>၂။ မဟုတ်ပါ။<br>၃။ မသိပါ။<br>၄။ မဖြေဆိုလိုပါ။
၄။	အထက်ပါ ထိခိုက်နစ်နာစေမှု(များ)ကြောင့် သင်စာသင်ချိန်များတွင် အာရုံစူးစိုက်ထားရန် (သို့) သင်ခန်းစာများကိုလွယ်လွယ်ကူကူနားလည် သဘောပေါက်နိုင်မှု ယခင်ကထက်ပိုမိုခက်ခဲလာသည်။	၁။ ဟုတ်ပါသည်။<br>၂။ မဟုတ်ပါ။<br>၃။ မသိပါ။

		၄။ မဖြေဆိုပါ။
၅။	အထက်ပါ ထိခိုက်နစ်နာစေမှု(များ)ကြောင့် မိမိကိုယ်ကို သေကြောင်းကြံရန် ပြီးခဲ့တဲ့ (၁၂) လ အတွင်း အလေးအနက် စဉ်းစားခဲ့ဖူးပါသလား။	၁။ စဉ်းစားခဲ့ဖူးပါသည်။<br>၂။ မစဉ်းစားခဲ့ဖူးပါ။<br>၃။ မသိပါ။<br>၄။ မဖြေဆိုလိုပါ။<br>(မစဉ်းစားခဲ့ဖူးပါကမေးခွန်း(၉)ကိုဆက်ဖြေပါ)

(ခ) အခြားသူများ၏ ထိခိုက်နစ်နာမှုများအား မျက်မြင်ကြုံတွေ့မှု (သင်ဖြေဆိုလိုသည့် အဖြေ၏ နံပါတ်ကို ဝိုင်းပါ။)

စဉ်	မေးခွန်း	အဖြေ
၁။	အထက်တွင်မေးမြန်းခဲ့သော ထိခိုက်နစ်နာစေမှုတစ်မျိုးမျိုး သင့်မိဘများအကြား ဖြစ်ပွားနေသည် ကို သင်မျက်မြင် ကြုံဖူး ပါသလား။	၁။ ကြုံဖူးပါသည်။<br>၂။ မကြုံဖူးပါ။<br>၃။ မသိပါ။<br>၄။ မဖြေဆိုလိုပါ။
၂။	အထက်တွင် မေးမြန်းခဲ့သော ထိခိုက်နစ်နာစေမှုတစ်မျိုးမျိုး သင့်သူငယ်ချင်းများအတွင်း ဖြစ်ပွားနေသည်ကို သင်မျက်မြင်ကြုံဖူးပါသလား။	၁။ ကြုံဖူးပါသည်။<br>၂။ မကြုံဖူးပါ။<br>၃။ မသိပါ။<br>၄။ မဖြေဆိုလိုပါ။
၃။	အထက်တွင်မေးမြန်းခဲ့သော ထိခိုက်နစ်နာစေမှုတစ်မျိုးမျိုး သင့် အိမ်နီးနာချင်းများတွင် ဖြစ်ပွား နေသည်ကို သင်မျက်မြင် ကြုံဖူးပါသလား။	၁။ ကြုံဖူးပါသည်။<br>၂။ မကြုံဖူးပါ။<br>၃။ မသိပါ။<br>၄။ မဖြေဆိုလိုပါ။
၄။	သင့်ပတ်ဝန်းကျင်၌ သင်မျက်မြင်ကြုံတွေ့ခဲ့ရသော ထိခိုက်နစ်နာစေမှု အမျိုးအစားများမှာ<br>(အဖြေတစ်ခုထက်ပို၍ ဖြေနိုင်ပါသည်။)	၁။ စိတ်ပိုင်းဆိုင်ရာ ထိခိုက်နစ်နာစေမှု<br>၂။ ရုပ်ပိုင်းဆိုင်ရာ ထိခိုက်နစ်နာစေမှု<br>၃။နည်းပညာအသုံးပြုထိခိုက် နစ်နာစေမှု<br>၄။ ကာယိန္ဒြေဆိုင်ရာ ထိခိုက်နစ်နာစေမှု<br>၅။ မသိပါ။<br>၆။ မဖြေဆိုလိုပါ။
၅။	အထက်တွင်မေးမြန်းခဲ့သော ထိခိုက်နစ်နာစေမှုတစ်မျိုးမျိုး ဖြစ်ပွား နေသည့် သတင်းကို သင်အသုံးပြုနေသော လူမှုကွန်ယက် များပေါ်တွင် သင်ကြားဖူးပါသလား။	၁။ ကြားဖူးပါသည်။<br>၂။ မကြားဖူးပါ။<br>၃။ မသိပါ။<br>၄။ မဖြေဆိုလိုပါ။
