# Supplementary material for: Assessing risk factors and impact of cyberbullying victimization among university students in Myanmar: A cross-sectional study
Source: PLoS One. 2020 Jan 22;15(1):e0227051. doi: 10.1371/journal.pone.0227051 (PMC6975531; doi:10.1371/journal.pone.0227051)
Supplement: S2 Questionnaire — (PDF) [file pone.0227051.s002.pdf]

Questionnaire regarding cyberbullying victimization:

(Please circle the number of your answer and/or write down in the blank.)

Code No. [ ]

I. Socio-demographic characteristics

A1	Age (completed years)	______________Years
A2	Sex	1. Male<br>2.Female
A3	Current Marital Status	1. Single<br>2. Married<br>3.Divorced/Separated<br>4. Widow
A4	How long have you been studied in this university?	_______________Years
A5	Do you live in dormitory?	1. Yes<br>2. No
A6	From which State/ Region do you come from?<br>(Permanent address)	_____________(State/Region)
A7	Name social media you use most commonly?	1. Facebook<br>2. Instagram<br>3. You tube<br>4. Viber<br>5. We Chat<br>6. Bee Talk<br>7. Other _________________<br>(Please specify.)
A8	How many average minutes/hours per day do you spend on that social media?	___________(minutes/hours)

II. Experiences regarding cyberbullying

	Did anyone ever	Answers
B1	hack or stalk or use your facebook/ social media account(s), or smart phone(s) without your consent and pretends to be you during the last 12 months?	1. Yes<br>2. No<br>3. Don't know<br>4. No response<br>(If not 'Yes', skip to B2)
B2	use your picture online without your permission during the last 12 months?	1. Yes<br>2. No<br>3. Don't know<br>4. No response<br>(If not 'Yes', skip to B3)
B3	tell lies or spread false rumours about you behind your back during the last 12 months?	1. Yes<br>2. No<br>3. Don't know<br>4. No response<br>If not 'Yes', skip to B4)
B4	send you humiliating/annoying/ mean texts or posts or sex chat during the last 12 months?	1. Yes<br>2. No<br>3. Don't know<br>4. No response<br>(If not 'Yes', skip to B5)
B5	make upsetting phone calls or malicious prank calls to you during the last 12 months?	1. Yes<br>2. No<br>3. Don't know<br>4. No response<br>(If not 'Yes', skip to B6)
B6	send unpleasant photos, sex pictures or videos to you against your will during the last 12 months?	1. Yes<br>2. No<br>3. Don't know<br>4. No response<br>(If not 'Yes', skip to B7)

B7	take your photo(s), video(s) without your consent or humiliate you or threaten you during the last 12 months?	1. Yes<br>2. No<br>3. Don't know<br>4. No response<br>(If not 'Yes', skip to B8)
B8	How old were you when you first experience this/these? (completed years of age)	_________Years
B9	Have you ever told anyone about this/these?	1. Yes<br>2. No<br>3. Don't know<br>4. No response

III. Adverse events following cyberbullying victimization

	Because of this/these cyberbullying events you suffered,	Answers
C1	You started/increased smoking cigarette.	1. Yes<br>2. No<br>3. Don't know<br>4. No response
C2	You started/increased betel chewing.	1. Yes<br>2. No<br>3. Don't know<br>4. No response
C3	You started/increased drinking.	1. Yes<br>2. No<br>3. Don't know<br>4. No response
C4	You become more difficult to concentrate on the lectures and to understand them easily than usual.	1. Yes<br>2. No<br>3. Don't know<br>4. No response
C5	Did you seriously consider attempting suicide during the last 12 months?	1. Yes<br>2. No

		3. Don’t know<br>4. No response

IV. Witnessing Psychological, Physical or Sexual Violence, or Cyberbullying in the neighbourhood

D-1	Have you ever witnessed any type of the violence mentioned above between your parents?	1. Yes<br>2. No<br>3. Don’t know<br>4. No response
D-2	Have you ever witnessed any type of the violence mentioned above among your friends?	1. Yes<br>2. No<br>3. Don’t know<br>4. No response
D-3	Have you ever witnessed any type of the violence mentioned above among your neighbours?	1. Yes<br>2. No<br>3. Don’t know<br>4. No response
D-4	Type of violence(s) you witnessed in your neighbourhood are	1. Psychological violence<br>2. Physical violence<br>3. Cyber violence<br>4. Sexual violence<br>5. Don’t know<br>6. No response
D-5	Have you ever heard news about any type of the violence/bullying mentioned above on social media you used?	1. Yes<br>2. No<br>3. Don’t know<br>4. No response
