# Supplementary material for: Assessing risk factors and impact of cyberbullying victimization among university students in Myanmar: A cross-sectional study
Source: PLoS One. 2020 Jan 22;15(1):e0227051. doi: 10.1371/journal.pone.0227051 (PMC6975531; doi:10.1371/journal.pone.0227051)
Supplement: Attachment — Submitted filename: Reviewer remarks, PLOS ONE.pdf [file pone.0227051.s003.pdf]

Manuscript Number: PONE-D-19-16609

Article Type: Research Article

Full Title: Assessing risk factors and impact of cyberbullying victimization among university students in Myanmar: A cross-sectional study

Short Title: Risk factors

THE PROS

The paper is written with clarity, referencing recent theoretical and valid empirical findings from authors who are relevant in the proposed field and topic of research.

The paper has a very interesting title, even though the sample does not only consist of typically adolescent participants (students) aged 18-25, but more mature participants, as well (27-35), which implies different cognitive, emotional and behavioral traits, so the authors could consider adjusting the title so as to emphasize this sample age difference.

The structure of the paper is satisfactory, but some of the elements or contents should be moved to different sections of the paper (e.g. the research procedure description, sample characteristics), so as to better suit the study presentation.

The study design is valid, the sample size and characteristics are satisfactory, while the chosen title and research area of the paper present a scientifically important subject, due to the scarcity of research findings on this topic.

THE CONS (recommendations for improvement)

A) Page 4 – ...„To surf the worldwide information technology tide....“ – this section feels disconnected from the rest of the section content, so it would be recommended inserting latest data findings from a meta-analysis by *Kowalski, R.M., Giumetti, G.W., Schroeder, A.N., Lattanner, M.R., 2014. Bullying in the digital age: a critical review and meta-analysis of cyberbullying research among youth. Psychol. Bull. 140, 1073–1137.* This would help the authors better explain the importance of some of the studied socioeconomic characteristics.

B) Page 5 - *Methods, Study area and participants* - The authors should explicitly state the age status of the sample, because the sample used is very diversified age-wise, while *age* has been found to be a very significant factor when studying cyberbullying among the adolescent population.

C) The instrument's methodological properties or metric characteristics need more thorough clarification. Was the instrument used in previous similar studies, and what are its methodological properties (validity, reliability)? How many questions or scales and what kind of questions were used, and how was the intensity or frequency measured (Likert scale, semantic differential, scale type...)? This is recommended, because the authors state that "*data were collected with a pre-tested, self-administered questionnaire. The questionnaire was developed by referring the domestic violence questionnaire from the 2015-2016 Myanmar Demographic and Health Survey [35], and the gender-based violence fact sheet published by the Department of Public Health, Ministry of Health and Sports, Myanmar [36].*"

D) Page 6 – It would be recommended to omit the company and address of the company that produced SPSS, as it is not usual and unnecessary in the paper.

E) Page 7 - The second part of the section *Ethical considerations* contains information that is better suited for the *Data collection* section of the paper, as it describes the procedure of the instrument administration and implementation, so it should be moved there.

F) It would be recommended the authors use the term „*Socioeconomic characteristics / status*“ or „*Socio-demographic characteristics / traits*“ instead of using the term „*Background characteristics*“, as it does not describe the studied variables correctly.

G) Page 8 - instead of using „*Magway*“ as a point of reference or variable name, I would suggest using the „*urban / rural*“ variable distinction when presenting and discussing results (e.g., Table 1., Background data).

H) *Table 1* should be revised so as to clearly show significant differences between the proposed variables (within the groups), as the significance is not visible in the table. For these kind of analyses, a *t-test* for independent samples (between 2 groups; e.g. marital

status) or *ANOVA* (3 groups or more; e.g. age group) would be recommended, as *chi-square tests* are usually implemented with smaller samples containing less than 30 participants.

I) The authors should decide on the style of data presentation, as it is uncommon to describe the results via text, and then provide the same data visually / graphically in a table (e.g. Table 2, page 9). It should be mutually exclusive, so the authors should decide what style of data presentation to use and implement it uniformly throughout the paper.

J) The notes presented under Table 2 (page 10) related to the types of cyberbullying should be presented in the *Introduction* / *Theoretical background* of the paper, where such definitions of cyberbullying and related behavior should be referenced and clarified.

K) *Table 3* is clear and presented well, but *Table 4* needs revision, as such a style of presenting regression analysis results is unusual. Table 4 is confusing and complex, as the data is not validly and clearly presented. Regression analysis should be presented using APA standards, with percentages of variance explained, and clear indicators of R^2, F for change in R^2, and R Square Change, as well as B, SE(B) and β coefficients as indicators of significant predictors. Maybe some examples from similar papers using the same type of statistical analysis would be helpful to the authors.

L) *Discussion*, page 14 - the authors reference studies on the relation of bullying and poor academic achievement, but it remains unclear whether the referenced studies report findings on traditional bullying or cyberbullying? ... „*Many studies have demonstrated that among students, bullying is strongly correlated with poor academic performance or outcomes [24,31-33]. Students who suffered peer bullying received lower grades and, faced academic difficulties and/or worsened academic performance compared with their non-bullied peers [24,25,31-33].*"

M) Considering the number of authors and their personal contribution to this paper, the *Discussion* section of the paper seems to be somewhat lacking, as it aims to describe only a segment of the results presented in the paper, while omitting study findings on various socio-economic characteristics that were established and presented in the authors' proposed study (risk behavior, suicide, use of substances, etc.). The authors

should revise and further explain all their results, as socioeconomic variables (aside from gender or age) were found in recent studies to be significant in explaining cyberbullying perpetration and victimization.

Goran Livazović
